# The Emerging Role of Induced Pluripotent Stem Cells as Adoptive Cellular Immunotherapeutics

**DOI:** 10.3390/biology12111419

**Published:** 2023-11-11

**Authors:** Vedika Mehra, Jyoti Bikram Chhetri, Samira Ali, Claire Roddie

**Affiliations:** Research Department of Haematology, Cancer Institute, University College London, Paul O’Gorman Building, London WCIE 6DD, UK

**Keywords:** induced pluripotent stem cells (iPSCs), chimeric antigen receptor T-cells (CAR-Ts), tumour-infiltrating lymphocytes (TILs), natural killer (NK) cells, virus-specific T-cells (VSTs), adoptive cell therapy (ACT), manufacturing, off-the-shelf, reprogramming

## Abstract

**Simple Summary:**

Adoptive cell therapy (ACT) is an innovative approach to combat cancer and infectious diseases using specialised immune cells, such as chimeric antigen receptor T-cells (CAR-Ts), tumour-infiltrating lymphocytes (TILs) and virus-specific T-cells (VSTs). Such therapies are manufactured individually for each patient and can be negatively affected by poor cell quality, often impaired by prior treatments, age, and complex manufacturing. To overcome this, this field is assessing the potential of creating cell therapies from “fit” donors to provide off-the-shelf treatment options. Induced pluripotent cells (iPSCs) have renewable characteristics and offer a solution towards off-the-shelf therapy. iPSCs can be used as an unlimited source to derive different immune cells, including natural killer (NK) cells and T-cells. iPSCs can be further genetically modified and used create different ACTs. In this review, we describe the methodologies for generating such cell therapies from iPSCs and discuss the current advances and challenges with a focus on CAR-T/NK-, TIL- and VST-based therapies.

**Abstract:**

Adoptive cell therapy (ACT) has transformed the treatment landscape for cancer and infectious disease through the investigational use of chimeric antigen receptor T-cells (CAR-Ts), tumour-infiltrating lymphocytes (TILs) and viral-specific T-cells (VSTs). Whilst these represent breakthrough treatments, there are subsets of patients who fail to respond to autologous ACT products. This is frequently due to impaired patient T-cell function or “fitness” as a consequence of prior treatments and age, and can be exacerbated by complex manufacturing protocols. Further, the manufacture of autologous, patient-specific products is time-consuming, expensive and non-standardised. Induced pluripotent stem cells (iPSCs) as an allogeneic alternative to patient-specific products can potentially overcome the issues outlined above. iPSC technology provides an unlimited source of rejuvenated iPSC-derived T-cells (T-iPSCs) or natural killer (NK) cells (NK-iPSCs), and in the context of the growing field of allogeneic ACT, iPSCs have enormous potential as a platform for generating off-the-shelf, standardised, “fit” therapeutics for patients. In this review, we evaluate current and future applications of iPSC technology in the CAR-T/NK, TIL and VST space. We discuss current and next-generation iPSC manufacturing protocols, and report on current iPSC-based adoptive therapy clinical trials to elucidate the potential of this technology as the future of ACT.

## 1. Introduction

Adoptive cell therapy (ACT) using chimeric antigen receptor (CAR) modified T-cells/natural killer (NK) cells, tumour-infiltrating lymphocytes (TILs) and virus-specific T-cells (VSTs) has revolutionised the treatment of a number of cancers and infectious diseases.

CARs are synthetic receptors designed to redirect T-cell or NK-cell effector functions against surface antigens found on cancer cells in a major histocompatibility complex (MHC) independent manner. The CAR structure consists of an extracellular single chain variable fragment (scFv) antigen-binding domain, an extracellular spacer region, a transmembrane domain and an intracellular signalling domain, comprising a T-cell receptor (TCR)-derived CD3z sequence and associated co-stimulatory domains [1]. CARs have been developed and tested against many cancer targets, but to date have demonstrated the greatest success within the clinic against CD19, a cell surface antigen which is highly expressed on B-cell-derived leukaemia and lymphoma. This led to the Food and Drug Administration (FDA) approval of four CD19-targeting CAR-T therapies for these indications, namely Tisagenlecleucel (Kymriah) [2], Axicabtagene ciloleucel (Yescarta) [3], Brexucabtagene autoleucel (Tecartus) [4] and Lisocabtagene maraleucel (Breyanzi) [5]. BCMA-targeting CARs have also been FDA-approved for use in multiple myeloma [6,7]. In contrast to chimeric antigen receptor T-cells (CAR-T), CAR-NK cells have not yet been FDA-approved but are the subject of ongoing clinical trials [8]. CAR NK cells have several potential biological advantages over CAR-Ts, namely a lack of MHC restriction, broader cytotoxic potential (via both the CAR and NK-specific innate pathways) and lower cytokine release upon antigen binding, potentially reducing the risk of cytokine release syndrome (CRS) and associated neurotoxicity frequently observed in patients undergoing CAR-T therapy [9]. 

In contrast to CAR therapies, TIL and VST therapies do not require a gene engineering step to recognise and bind target antigens; rather, they utilise the endogenous TCR to direct cytotoxicity against cancer and viral infection, respectively. Whilst progress towards the licensing and widespread application of TIL and VST therapeutics has been less rapid than for CAR-T therapy, these products have shown impressive responses against a range of solid tumours [10,11] and viral infections [12,13].

CAR-T/NK manufacture requires patient or healthy donor leukapheresis (in some cases, cord blood can be used), viral vector transduction and cell expansion, and the manufacturing time varies from <10 days to >30 days [14,15,16]. TIL/VST manufacture is often more time-consuming and complex due to the low frequency of initiating tumour/virus-reactive T-cells obtained from the starting material, and as such, manufacture can take between 5 and 10 weeks per product [17,18]. TIL manufacture requires the fragmentation or enzymatic digestion of excised patient-derived tumours, the enrichment of tumour-reactive TILs through IFN-γ-based selection following stimulation with autologous tumours, and a rapid expansion protocol (REP) in the presence of IL-2 and anti-CD3 antibodies to achieve target cell doses for patient treatment [17,19]. As an alternative to IFN-γ-based selection, other groups have explored the use of PD-1 (CD279) and 41BB (CD137) T-cell activation marker-based selection to enrich more specifically for tumour-reactive TILs [11,20,21]. For further stringent tumour-specific TIL enrichment, whole-exome sequencing and improvements in the generation of tandem minigene (TMG) libraries permit the identification of neoantigen-specific TILs for downstream expansion [11,17]. These products are currently being tested in clinical trials [22]. To expedite TIL manufacture, some groups have eliminated the IFN-γ-based TIL enrichment step, instead proceeding directly to a REP following tumour fragmentation/digestion [23,24]. 

VSTs can similarly be manufactured through T-cell expansion following direct viral peptide stimulation or co-culture with peptide-pulsed antigen-presenting cells (APCs), where VSTs can be isolated and enriched using multimer and IFN-γ-based selection [18].

Whilst ACTs have demonstrated impressive results within the clinic, there are several formidable challenges in the widespread delivery of products to the number of patients who need them. Firstly, the majority of ACT products are autologous therapies, bespoke to each patient, which increases cost and limits product standardisation, mainly due to patient-specific factors such as age-related immunosenescence, prior chemotherapy and underlying disease. Secondly, lengthy manufacturing times often result in delayed patient treatment, leaving patients vulnerable to disease progression and death before they can receive the product [25,26,27]. For these reasons, allogenic ACT approaches are gaining traction in what is a growing effort to generate a truly standardised universal ACT, manufactured at scale and batched for all patients [28,29,30]. Frequently, healthy donor T-cells are used as the starting material for allogeneic ACT products, and gene editing of the TCR and other cell surface molecules is often required to reduce the risks of graft-versus-host disease (GvHD) and graft rejection, respectively [29,30,31]. Despite clear potential advantages of the allogeneic approach, the manufacture of off-the-shelf therapies can be complex and expensive, requiring multiple healthy donors for each batch of ACT product, with a requirement to repeat the donor harvest and manufacture process when each batch is exhausted [29,30]. 

The ability to generate induced pluripotent stem cells (iPSCs) from any somatic cell through the introduction of selective transcription factors paved the way for groundbreaking scientific advances. iPSCs harbour self-renewal capabilities, accompanied with the potential to differentiate into cell types of all three germ layers, making them an invaluable resource for use in disease modelling and cellular therapeutics. Such characteristics of iPSCs similarly offer potential solutions to some of the problems with allogenic ACTs described above. As a renewable cell source, iPSCs eliminate the requirement for multiple healthy donor-derived batches. iPSCs have been successfully shown to generate functional NK/T-cells [32,33] and, in the ACT space, have the distinct advantage of limitless clonal replicative potential, which can be coupled with gene-editing approaches to provide a standardised source of NK/T-cells [34,35]. Through this review, we will explore the use of iPSCs in CAR, TIL and VST ACT manufacturing.

## 2. Discovery, Generation and Characterisation of iPSCs

In 2006, Shinya Yamanaka made a revolutionary scientific advancement by successfully reprogramming mouse and human somatic cells into iPSCs [36,37]. Such reprogramming was initially achieved by introducing a combination of four reprogramming transcription factors, namely Oct 3/4, Klf4, Sox2 and c-Myc, known as “OKSM-Yamanaka factors” into adult human fibroblasts. The resulting iPSCs displayed marked similarities to human embryonic stem cells (ESCs) in relation to their morphology, proliferation rate, surface antigens, gene expression, epigenetic profiles of pluripotency-related genes, telomerase activity and their ability to differentiate into cell types of the three germ layers in vitro and form teratomas in vivo [36,37]. This breakthrough allowed for the reprogramming of differentiated cells back to a pluripotent state, bypassing the ethical concerns associated with the derivation of ESCs derived from the inner cell mass of a blastocyst. Subsequent studies showed that Klf4 and c-Myc can be replaced by the transcription factors L-Myc [38], Nanog and Lin28 [39] for the efficient reprogramming of human somatic cells. Methods eliminating the use of c-Myc and Lin28 are thought to reduce neoplastic risk associated with iPSC reprogramming [40]. iPSCs can be successfully generated from a range of somatic cells including cord blood [41], dermal fibroblasts [42] and peripheral blood mononuclear cells (PBMCs) [43]. With their potential to differentiate into various cell types, the use of iPSCs is invaluable for disease modelling, drug screening and tissue regeneration, and has opened new avenues in regenerative medicine and personalised therapeutics.

Efficient reprogramming relies on the delivery of OKSM/Nanog/Lin28 transcription factors via integrating or non-integrating approaches. Integrating approaches using retroviral or lentiviral vectors are highly efficient gene transfer methods, permitting stable transgene expression, but the random nature of the integration event into the host genome carries a risk of insertional mutagenesis and oncogene-activation-led tumorigenesis [44]. To avoid this, transcription factor delivery using non-integrating viral vectors, such as Sendai virus [38,45] and adeno-associated virus [46], and other non-integrating approaches, such as episomal plasmids [47,48], messenger RNA [49], transposon [50] and minicircle [51]-based methods have been developed. Such non-integrating methodologies offer a cGMP clinically applicable method of iPSC generation.

Following the reprogramming of somatic cells, iPSCs must undergo stringent characterisation to ensure pluripotency and genome stability. Initially, emerging iPSCs can be identified using an alkaline phosphatase (AP) live stain (high expression in ESCs/iPSCs), followed by the characterisation of pluripotent colonies using surface (TRA-1-60, TRA-1-81, SSEA3, SSEA4) and intracellular (Oct3/4, Sox2, Nanog) staining via flow cytometry or immunofluorescence microscopy [52]. iPSCs can then be maintained under lineage-specific culture conditions and tested for their ability to generate all three germ layers using markers specific for the ectoderm (PAX-6, Nestin), endoderm (CXCR4, Sox17, FOXA2) and mesoderm (Brachyury, NCAM). Trilineage differentiation can further be identified through teratoma formation following iPSC injection into immunocompromised mice [53]. Lastly, genome stability can be confirmed through G-banding karyotyping, whole-genome sequencing (WGS) and single-nucleotide polymorphism (SNP) arrays to ensure the absence of chromosomal aberrations, point mutations and copy number variations. This is common in iPSCs due to existing aberrations in somatic cells, or aberrations introduced by reprogramming or long term culture [44,54].

## 3. PBMC Reprogramming

The optimisation of reprogramming efficiency was initially conducted in fibroblasts, which are adherent cells with a high long-term proliferative capacity, i.e., characteristics that favour reprogramming. Whilst efficiency is largely dependent on the reprogramming method used, fibroblasts have shown average reprogramming efficiencies of >0.01% [46,55,56] (range, 0.0002%–>10%). Whole PBMCs or isolated B-cells, CD34+ cells and T-cells can be reprogrammed as mixed or independent populations. The reprogramming of PBMCs is less efficient than that of fibroblasts (on average, <0.001% (ranging from <0.0001% to 0.1%), which is in part due to them being suspension cell cultures and thus vulnerable to cell loss during the frequent media changes required during reprogramming. The serial seeding of cord blood-derived PBMCs onto vitronectin-coated plates with centrifugation has been shown to minimise cell loss and increase iPSC yield [43]. 

With a focus on PBMCs, T-cells require specific consideration of their differentiation status prior to iPSC reprogramming. To increase reprogramming efficiency, studies have shown that T-cell activation is critical [57], using methods such as plate-bound/soluble CD3/CD28 antibodies [57,58], Phytohaemagglutin (PHA) and Dynabeads^TM^ [32]. Additionally, T-cells harbour a range of differentiation states, including naïve T (Tn), central memory (Tcm), effector (Te) and terminal (Tte) subsets, with a progressive loss of proliferative potential [59]. Strong or protracted T-cell activation can drive T-cell differentiation towards Te/Tte-enriched subsets, such as those commonly observed in TIL and VST cultures. Reprogramming Te/Tte subsets is challenging due to activation-induced cell death (AICD) and reduced proliferative potential, but feasibility has been demonstrated using Sendai virus vectors encoding OKSM [32,57,58]. In one study, peripheral blood-derived Tte cells activated with plate-bound CD3 antibodies were able to generate iPSCs, with efficiencies up to 0.1%, with increasing virus multiplicity of infection (MOI) [58]. Similarly, following two consecutive rounds of CD3/CD28 antibody stimulation, melanoma-derived TILs generated iPSCs at efficiencies of 0.01–0.05%. In parallel, control PBMCs with a greater Tcm (CCR7+) profile generated iPSCs at an efficiency of 0.1% [57]. Further, a range of antigen-specific T-cells targeting HIV-1, Nef, CMV pp65, GAD and α-GalCer were successfully reprogrammed following Dynabead^TM^ and PHA stimulation at lower efficiencies from 0.000002% to >0.003% [32].

**Figure 1:**
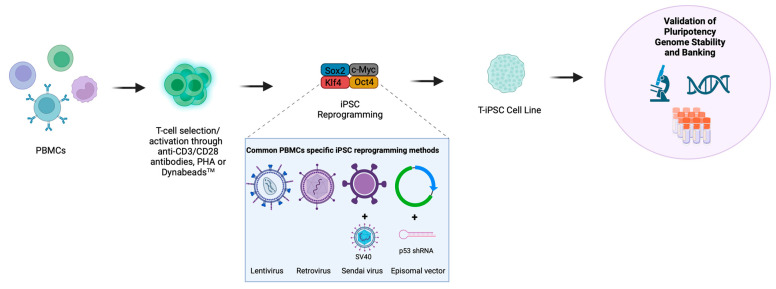
**Methods of T-cell reprogramming.** T-cells can be reprogrammed directly from bulk peripheral blood mononuclear cells (PBMCs) or following a T-cell selection step. T-cells are primarily activated via anti-CD3/CD28 antibodies, Phytohemagglutinin (PHA) or Dynabeads^TM^, and subsequently reprogrammed into iPSCs through the introduction of transcription factors, which most commonly include, OCT4, SOX2, KLF4 and MYC (OSKM). The most common methods for the delivery of OSKM transcription factors into T-cells include the use of lentivirus, retrovirus, Sendai virus or episomal vectors where Sendai virus and episomal vectors have been supplemented with SV40 large T antigen or a p53-targeting shRNA, respectively. The resulting T-cell-derived iPSC colonies (T-iPSCs) must undergo stringent characterisation to confirm pluripotency and genome stability prior to banking and use in downstream differentiations. Created with BioRender.com.

Senescent cells lacking proliferative ability are more likely to fail reprogramming [60]. The transduction of these cells with SV40 large T antigen (the function of which is to disable retinoblastoma (Rb) and p53 tumour suppressor pathways towards enhanced proliferation [61]) or short hairpin RNA (shRNA) against p53 [62] alongside the OKSM factors [32] can improve reprogramming efficiency. Similarly, episomal plasmid-based PBMC reprogramming with Oct3/4, Sox2, Klf4, L-Myc, Lin28 and shRNA against p53 and EBNA1 protein (to permit the high and persistent expression of transcription factors) resulted in reprogramming efficiencies of 0.1% [47]. A summary of the PBMC/T-cell reprogramming methods is illustrated in Figure 1. 

## 4. Methods of iPSC-to-T-Cell Differentiation

**Figure 2:**
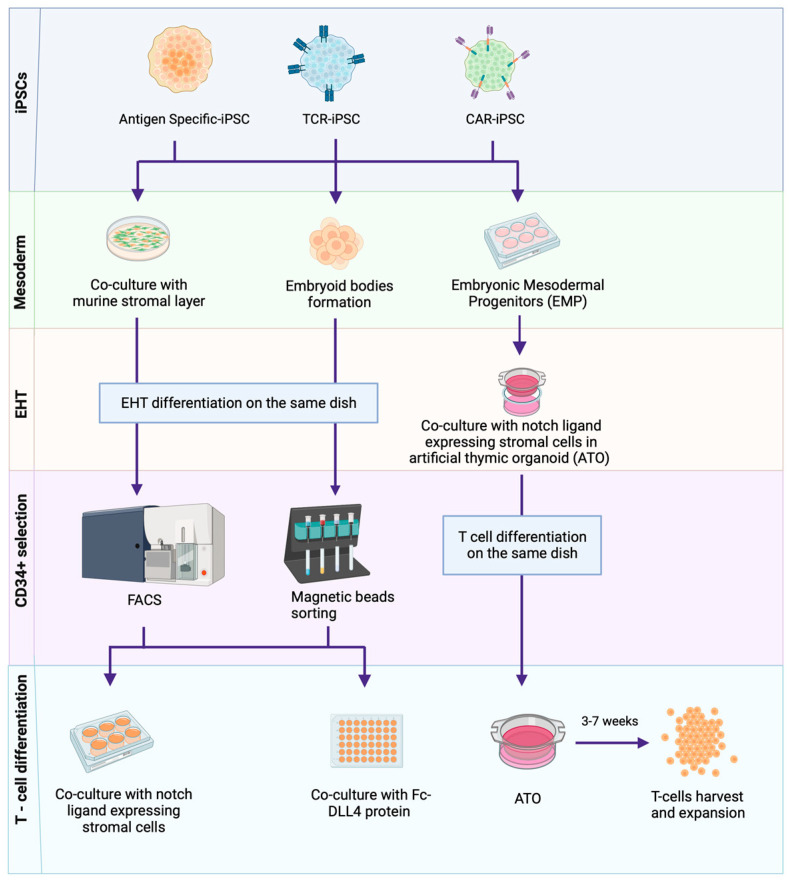
**Methods of iPSC–to–T-cell differentiation.** T-cell-derived iPSCs are guided through a multi-step redifferentiation process to acquire antigen-specific T-cells. This process starts with the differentiation of iPSCs into mesoderm lineages and subsequent endothelial-to-hematopoietic transition (EHT), achieved through various methods, including co-culturing iPSCs with murine stromal layers (OP9 or C3H10T1/2), the formation of stroma-free embryoid bodies (EBs) and the use of cytokine cocktails to obtain hematopoietic stem cells (HSCs) and embryonic mesoderm progenitors (EMPs). Upon the induction of HSCs, T-cell differentiation is facilitated through methods including co-culture with murine stromal layers (OP9-DLL1/4), the use of Fc-DLL4 (Delta like ligand 4) protein or through a 3D artificial thymic organoid (ATO) system. A cultivation period of approximately 3–7 weeks results in the production of expanded T-cells. Created with BioRender.com. Key: Antigen-specific T-cell-derived iPSCs (Antigen-specific-iPSCs), T-cell receptor transduced iPSCs (TCR-iPSCs), Chimeric antigen receptor knock-in/transduced iPSCs (CAR-iPSCs).

Figure 2 summarises the iPSC-to-T-cell differentiation steps. The generation of T-cells from iPSCs is a lengthy (35 days/longer) two-step process comprising the generation of haematopoietic stem cells (HSCs) and subsequent T-cell lineage commitment [32,63,64,65,66,67,68]. HSC generation can be achieved through a number of methods involving the co-culture of iPSCs with mouse embryonic stromal cell lines (e.g., OP9 [63,64,65] or C3H10T1/2 [32,66]) or without stromal cells through embryoid body (EB) formation on low-attachment plates under variable media and cytokine compositions [67,68,69]. HSCs (CD34+) are then committed to T-cell lineage following the activation of Notch signalling, which plays a critical role in haematopoiesis [70]. OP9 cells, engineered to express the notch ligands delta-like ligand 1 (DLL1) and the more potent DLL4 under a range of media and cytokine compositions, can successfully generate mature T-cells from iPSCs [71,72]. Some studies suggest that DLL4 is better for the generation of T-cells [73,74], but this may vary depending on the parental iPSC source. T-cell-derived iPSCs (T-iPSCs) show early T-cell receptor (TCR) expression and may require more potent notch signalling with DLL4, whereas DLL1 may be sufficient for ESCs, fibroblast-derived iPSCs and T-iPSCs with TCR knockout [69]. 

Alternative T-cell differentiation methods to OP9-DLL1/4 include the use of murine foetal thymic lobes and artificial thymic organoids (ATOs). In the foetal thymic lobe setting, iPSC-derived immature T-cells cultured on OP9-DLL1 stromal cells are seeded in a hanging drop plate 3D culture with murine foetal thymic lobes [75]. In this ATO system, mesoderm induction is initiated in feeder-free conditions under a cytokine cocktail, where human embryonic mesoderm progenitors (hEMPs) (CD326−/CD56+) are aggregated into organoids using the MS5-DLL4 mouse stromal cell line on a porous membrane [76]. 

For clinical applicability, differentiation methods that do not require murine stromal cell lines, namely the addition of exogenous DLL4 protein, have been developed. Here, iPSC-derived CD34+ cells are cultured with DLL4-coated streptavidin polystyrene beads [77], or EB-derived CD34mid/CD43+ populations are cultured on DLL4 protein-coated retronectin plates. This DLL4 protein-based method has been shown to be highly efficient, such that 3 × 10^5^ iPSCs can generate 6.2 × 10^8^ CD8αβ+ T-cells [68]. 

Directly comparing the differentiation efficiency of each method is difficult, confounded by the different culture conditions used between studies, as summarised in Table 1. Most protocols demonstrate progressive T-cell commitment akin to that seen during native T-cell development in the thymus. At the outset, CD4−/CD8− double-negative (DN) cells predominate, but progressive differentiation leads to CD4+ intermediate single-positive (ISP) populations and then CD4+/CD8+ double-positive (DP) states. To generate CD8αβ+ single-positive T-cells from CD4+/CD8+ DP subsets, most protocols incorporate a T-cell activation step using CD3/CD28 antibodies/PHA/peptide simulation or co-culture with antigen presenting cells (APCs) engineered with costimulatory domains (e.g., 4-1BB) [32,64,65,66,67,68]. 

The emergence of erroneous CD8 T-cell populations has also been reported during iPSC-to-T-cell differentiation. Whilst the classical emergence of CD8αβ+ T-cells, representing mature T-cells, is desired, CD8αα+ homodimers have been reported. These homodimers are classically found on innate immune cells with impaired TCR signalling and strong TCR-independent cytotoxicity [67,76,78]. A study attributed the stimulation of CD4−/CD8− DN subsets to the generation of CD8αα+ T-cells, whereas the stimulation of CD4+/CD8+ DP subsets gives rise to CD8αβ+ [79]. Some iPSC-derived T-cells have further been shown to lack T-cell-specific surface antigens such as CD2/CD28/CD5, and have a high expression of innate cell markers such as CD56 [32,67]. 

Current protocols convincingly demonstrate that functional CD8 T-cells can be derived from iPSCs. To date, there is limited data to guide iPSC differentiation into CD4 T-helper subsets, with the exception of the ATO method, which has demonstrated the differentiation of iPSCs into functional CD4+ SP T-cells, productive of cytokines, such as IL-2/IFN-γ/IL-4 [76]. The incorporation of gene editing in the ATO system to knock out TBX21 (encoding T-bet) or IL4 (Th2 regulator) genes in iPSCs resulted in the generation of CD4 SP T-cells, exclusively productive of IFN-γ and IL-4, recapitulating Th1 and Th2 subsets, respectively [80]. 

**Table 1:** Summary of reported primary culture components used for HSC induction, and T-cell differentiation, maturation and expansion from iPSC cell lines across studies.

**Mesoderm/HSC Induction**			
**Methods**	**Embryoid Bodies**	**Co-Culture with OP9/C3H10T1/2**	**Artificial Thymic Organoids (ATOs)**
Primary Culture Components	CHIR99021 10 µM	VEGF (15–50 ng/mL)	rhActivin A (10 ng/mL)
	SB431542 6 µM	SCF (50 ng/mL)	rhBMP4 (10 ng/mL)
	BMP-4 (10–50 ng/mL)	FLT3L (10 ng/mL)	rhVEGF (10 ng/mL)
	bFGF (5–50 ng/mL)		rhFGF (10 ng/mL)
	VEGF (15–50 ng/mL)		
	FLT3L (10 ng/mL)		
	SCF (50 ng/mL)		
	TPO(30 ng/mL)		
References	[67,68,74]	[32,65,81]	[76]
**T-cell differentiation**			
**Methods**	**Fc-DLL4/Retronectin** **(stroma free)**	**Co-culture with OP9−DLL1/4**	**Artificial thymic organoids (ATOs)**
Primary Culture Components	rhFLT-3L (50 ng/mL)	rhFLT-3L (5–10 ng/mL)	rhFLT-3L (5 ng/mL)
	rhIL-7 (50 ng/mL)	rhIL-7 (1–10 ng/mL)	rhIL-7, first 7 days (5 ng/mL)
	rhSCF (50 ng/mL)	rhSCF (5–10 ng/mL)	rhSCF, first 7 days, (10 ng/mL)
	rhTPO (100 ng/mL)		rhSCF, after 7 days (50 ng/mL)
	rhSDF-1α (30 nM)		rhTPO, after 7 days, (5 ng/mL)
	SB203580 (15 µM)		
References	[68]	[32,65,66,67,79,81,82]	[76]
**T-cell Maturation/Expansion**			
**Methods**	**anti-CD3/CD28 mAbs**	**anti-CD3**	**Phytohemagglutinin (PHA)**
Primary Culture Components	hIL-7 (2–5 ng/mL)	hIL-7 (10 ng/mL)	hIL-7 (10 ng/mL)
	hFlt-3L (0–5 ng/mL)	rhIL-2 (10 ng/mL)	IL-15 (5 ng/mL)
	hSCF (0–10 ng/mL)	dexamethasone 10 nM	PHA 2 μg/mL
	anti-human CD3 (50–5000 ng/mL)	Anti-human CD3 (OKT3) 500 ng/mL	
	anti-human CD28 (1000–2000 ng/mL)		
	hIL-2 (2 ng/mL/200 U/mL)		
References	[74]	[68]	[64,82,83]

## 5. Methods of iPSC-to-NK Cell Differentiation

**Figure 3:**
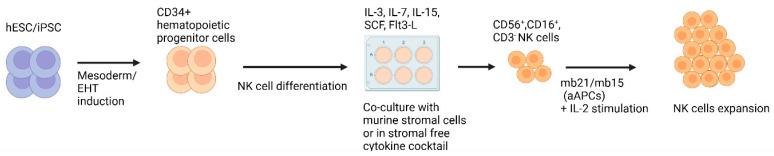
**Methods for generating iPSC−derived NK cells.** This figure illustrates the methods employed to derive NK cells from iPSCs. First, hematopoietic cells are produced from iPSCs. HSCs are subsequently cultivated on murine stromal cells or in a stromal-free cytokine cocktail for the development of NK cells, commonly characterised by the presence of CD56+, CD16+ and CD3− markers. Generated NK cells can undergo additional expansion through co-culture with artificial antigen presenting cells (aAPCs) engineered to express membrane-bound (mb) IL-21/IL-15 in the presence of IL-2. Created with BioRender.com.

Similar to T-cells, NK cells can also be successfully derived from iPSCs, and the process is outlined in Figure 3. Initial HSC generation is similar to T-cell protocols where mouse stromal cells such as M2-10B4/S17 and spin EB generation have been used [83,84]. Unlike T-cells, differentiation into NK cells does not require Notch signalling, but instead requires specific cytokine cocktails and mouse bone marrow stromal cell lines, such as AFT024 and EL08-1D2 [83,85]. 

In parallel, cGMP protocols towards NK cell differentiation have been developed for clinical application that eliminate serum- and mouse-derived stromal cells [83]. Robust NK cell expansion is then achieved using exogenous IL-2 and 41BB/membrane-bound IL-21 engineered APCs [83,86,87]. Membrane-bound IL-21 can expand NK cells by 60-fold and is superior to membrane-bound IL-15 [87]. Phenotyping demonstrates the expression of mature NK cell markers (CD56, CD16, CD94), NK activating receptors (NKG2D, DNAM-1), NK cytotoxic receptors (NKp46, NKp44, KIRs) and cell death ligands (FasL, TRAIL) [83,84,85,88]. iPSC-derived NK cells demonstrate cytotoxicity through cytokine/chemokine, death receptor and antibody-dependent cellular cytotoxicity (ADCC) mechanisms [89].

## 6. Use of iPSCs in Adoptive Cell Therapy

### 6.1. CAR T-Cell Therapy

One early example of iPSC-derived CD19-targeting CAR-T therapy was developed by Themeli et al. [67], who showed that T-iPSC cell lines can be transduced with a CAR cassette using lentiviral vectors, and CAR-T-iPSC cell lines can then be successfully differentiated into CAR-Ts. The resulting products were CD8αα+, which lacked CD5 and expressed the NK marker CD161, matching a phenotypic profile commonly seen with (innate) γδ T-cells. The CAR-Ts elicited robust anti-tumour responses in an in vivo Burkitt’s Lymphoma murine model [67]. 

The potential benefits of allogeneic iPSC-derived CAR-T therapies include the development of standardised products and rapid access to an off-the-shelf product for patients. A substantial risk of allogenic T-cell therapy is the risk of human leukocyte antigen (HLA) mismatch between the donor/recipient leading to graft rejection or fatal GvHD. A potential mitigation strategy is to bank multiple HLA homozygous CAR-iPSC cells lines, such that HLA-typed patients could receive “best-matched” CAR-iPSCs. In terms of scale, a bank of 50–175 iPSC lines has been estimated to be sufficient to cover >70% of Japanese and UK patient populations, respectively [89,90,91]. 

To mitigate for the risk of GvHD from an iPSC-derived allogeneic CAR product, an alternative strategy to the HLA-diverse CAR-iPSC bank approach outlined above is to use iPSC gene editing to delete the TCR. A particular advantage of iPSCs is that their clonal growth characteristics permit the stringent selection of muti-edited cell lines, even at a low editing efficiency [92]. Multiple edited clones can be further screened for genome stability and off-target genomic toxicity to improve the safety profile of resulting iPSC-CAR products. CRISPR Cas9 has been used for multiplex iPSC editing to not only delete the TCR, but to delete the HLA-I and HLA-II genes and thus reduce the risk of immunological rejection and GvHD. However, additional modifications were added to prevent NK-mediated rejection, which has been reported when HLA is disrupted, namely the knockout of the NK-activating receptor DNAM-1 and transduction with HLA-E to inhibit NK-mediated lysis. T-cells differentiated from this multi-edited iPSC cell line demonstrated immune escape against allogeneic immune cells in vitro and in vivo [34]. 

With the goal of preventing GvHD and inducing more physiological expression of CAR (akin to the expression of native TCR), a further study of CAR-iPSCs utilised CRISPR-mediated homology-directed repair (HDR) to insert a CD19-targeting CAR construct [93] into the T-cell receptor α constant (TRAC) gene locus encoding TCR. This simultaneously disrupts TCR expression whilst inducing CAR expression under the control of the physiological TCR promoter. These edited iPSC cell lines were able to derive functional CD8αβ+ CAR T-cells with anti-tumour activity in an in vivo leukaemia model without inducing GvHD in mice [69]. 

Another study assessed the CRISPR-mediated knockout of diacylglycerol (DAG) isoforms α/ζ to enhance RAS/ERK signalling in a glypican-3 (GPC-3)-targeting CAR iPSC cell lines, and the resulting CAR-iPSCs underwent retroviral transduction with membrane-bound IL-15/IL-15α. These edits were found to enhance anti-tumour responses in comparison to non-edited iPSC-derived CAR T-cells [66]. 

**Figure 4:**
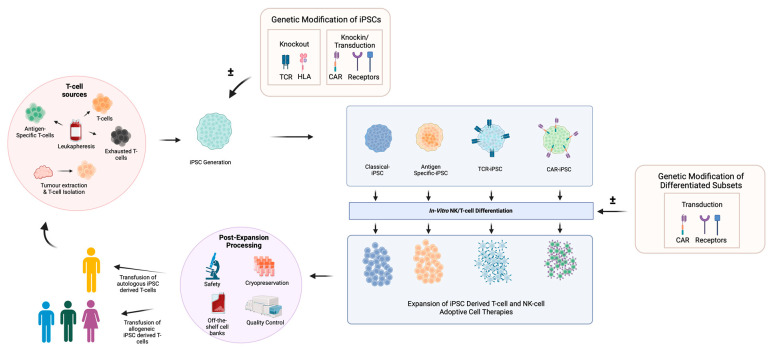
**Generation of adoptive cell therapies from iPSCs.** T-cell subsets including antigen-specific T-cells and exhausted T-cells, which can be obtained from patient leukapheresis and tumour-infiltrating lymphocytes (TILs), are sourced following tumour dissociation. These diverse T-cell populations are reprogrammed into iPSCs and tailored through genetic modification to induce the knockout of genes such as T-cell receptors (TCRs) or human leukocyte antigens (HLAs) to mitigate alloreactivity. Elements such as chimeric antigen receptors (CARs), tumour antigen-specific TCR receptors and the HLA-E complex can be introduced for anti-tumour functionality and to mitigate graft rejection. The generated classical/modified iPSC cell lines undergo NK/T-cell-specific differentiation, where resulting cells can be further modified to augment functionality. For use as cell therapeutics, derived products must be expanded to sufficient treatment doses and cryopreserved. Rigorous safety and quality control assessments are required prior to clinical treatment where differentiated T-cells can then be infused as allogeneic or autologous T-cell therapies. This process can be utilised to generate a variety of CAR T-cell, CAR NK, TIL and VST ACTs. Created with BioRender.com. Key: Unmodified T-cell-derived iPSCs (Classical iPSCs), Antigen-specific T-cell-derived iPSCs (Antigen-specific-iPSCs), T-cell receptor transduced iPSCs (TCR-iPSCs) and Chimeric antigen receptor knock-in/transduced iPSCs (CAR-iPSCs).

Together, these studies demonstrate that the multiplexed gene editing of iPSCs to generate allogeneic CAR-T products is feasible. Clinical testing is underway, with Fate Therapeutics leading a phase I dose escalation of iPSC-derived CD19 CAR T-cells where the CAR is traduced into a TRAC locus (FT819) against B-cell lymphoma and leukaemia. The interim results demonstrate safety and anti-tumour activity [94]. Other CAR-iPSC T-cell therapies in clinical development are highlighted in Table 2, and a summary of the use of iPSCs in adoptive cell therapy is illustrated in Figure 4.

### 6.2. CAR NK Cell Therapy

Due to the lack of MHC restriction and the ease of NK cell generation from iPSCs, iPSC-derived CAR NK therapy is an attractive allogenic treatment option. Early studies of CAR NKs utilised the same CAR design as T-cells [95], but Li et al. [96] subsequently showed that using NK-specific activation and co-stimulatory domains could improve the efficacy of CAR-NKs in vitro and in vivo. Specifically, combining the NKG2D transmembrane domain, the 2B4 co-stimulatory domain and the CD3z signalling domain resulted in powerful activation and anti-tumour responses from iPSC-derived CAR-NKs in an in vivo ovarian cancer model. The iPSC-derived CAR-NK cells demonstrated anti-tumour activity in vivo, in keeping with classical CAR T-cells, but with less toxicity [96]. Fate Therapeutics are paving the way for the use of iPSC-derived NK cells, having initiated numerous trials testing both classical iPSC-derived NK cells (FT500) [97] and NK cells with function-enhancing edits against a range of cancers. The iPSC cell lines used to derive the NK cell therapies are edited with a non-cleavable CD16 module that can bind to the Fc portion of co-infused anti-tumour monoclonal antibodies to enhance ADCC (FT516) [98]. Others edits include the addition of non-cleavable CD16 with an IL-15 receptor fusion to enhance persistence, the addition an anti-CD19 CAR NK-specific construct (FT596) [35] and the knockout of CD38 to limit CD38-mediated fratricide when given in combination with a CD38-targeting antibody (FT536/FT576) [99,100]. Trial results from FT516 against a range of solid tumours in combination with Avelumab, as well as FT596 against B-cell lymphoma in combination with Rituximab, demonstrate efficacy and a favourable safety profile [35,98]. All trials incorporating iPSC-derived NK cell therapy are listed in Table 2, and a summary of the use of iPSCs in adoptive cell therapy is illustrated in Figure 4.

### 6.3. TIL Therapy

TIL therapy relies on anti-tumour TCR specificity, and an important consideration in the TIL-iPSC field is whether T-cells reprogrammed to iPSCs faithfully retain the same TCR when differentiated back into T-cells. During T-cell development in the thymus, TCRβ and TCRα genes are rearranged between the late CD4−/CD8− DN and the CD4+/CD8+ DP stage through recombination, activating genes RAG1 and RAG2 [101]. Studies have demonstrated that MART-1 [75] and LMP2 [79] epitope-specific, antigen-specific T-cells could be reprogrammed into iPSC cell lines and re-differentiated into CD8 T-cells with retained TCR specificity. Whilst 90% of iPSC-derived re-differentiated MART-1 T-cells retained the original epitope [75], a 4.6% proportion of iPSC-derived re-differentiated LMP2 CD8 T-cells lost epitope specificity following stimulation, attributed to TCRα rearrangement [79]. Subsequently, it has been demonstrated that TCR specificity can be stabilised through the knockout of the RAG protein complex required for TCRα/TCRβ rearrangement. As an exemplar, iPSCs were generated from GPC-3-specific CD8 T-cells and RAG was knocked out at the iPSC stage using CRISPR. Differentiated CD8 T-cells retained GPC-3 TCR specificity in RAG knockout iPSCs, whereas wild-type RAG-derived T-cells lost 40% of their antigen specificity [81]. Similar TCR stability was seen following RAG knockout in monocyte-derived iPSCs transduced to express TCRs against GPC-3 and WT1. The resulting differentiated CD8 T-cells demonstrated preserved monoclonal expression of the transduced TCRs [81]. 

Whilst technically promising, studies primarily demonstrate feasibility in antigen-specific T-cells; however, these are single-antigen-targeting treatments restricted by HLA, thus limiting the number of patients that can be treated. True TIL therapy would require the autologous generation of polyclonal tumour-reactive T-cells from iPSCs. The feasibility of such a process was demonstrated by Ito et al., where TILs isolated from colorectal tumours were expanded and co-cultured with autologous tumour spheroids. Reactive TILs were sorted based on CD107a/41BB markers where TCR specificity was confirmed through HLA class 1 blocking; these TILs were subsequently reprogrammed to iPSCs. Using a feeder-free protocol, functional multiclonal tumour-specific CD8 T-cells were generated with the absence of additional TCR rearrangements [102]. Although feasible, a key limitation of utilising iPSCs in autologous TIL therapy is the reprogramming efficiency. Current protocols are low in efficiency, which greatly narrows the TCR repertoire that can be achieved. Moreso, the manufacturing time and costs, as well as the ability to quality control such a bespoke therapy, will inform its potential as a clinical therapeutic. A summary of the use of iPSCs in adoptive cell therapy is illustrated in Figure 4.

### 6.4. VST Therapy

Like TIL therapy, VST therapy relies on TCR specificity. Researchers have similarly demonstrated that iPSC cell lines can be generated from virus-specific T-cells and differentiated back into functional CD8 T-cells with retained TCR specificity in HIV [32], human papilloma virus type 16 (HPV16) [103] and Epstein–Barr virus (EBV) [79,104] models. Apart from HIV, HPV16- and EBV-specific T-cells have been investigated in the context of HPV/EBV-associated cancer [32,79,103,104]. Whilst Nishimura et al. demonstrate that Cytomegalovirus (CMV)-reactive T-cells specific for the pp65 CMV antigen could be successfully reprogrammed [32], the use of iPSCs in classical VST therapy against CMV, EBV and Adenovirus (Adv) has not been studied. Adoptive cell therapy against viral infection can be used to clear infection or as a prophylactic against viral reactivation in immunocompromised individuals, and has shown remarkable efficacy. Manufacturing such VSTs can take several weeks and involves the expansion of VSTs through direct stimulation with pooled viral peptides or simulation with peptide-pulsed/viral transduction of dendritic cells with virus-specific antigens. VSTs can be manufactured as both autologous and allogeneic therapies [18]. In the allogenic setting, it is suggested that VSTs with defined TCR specificity eliminates alloreactivity, leading to GvHD [105]. This requires the stringent selection of viral antigen-reactive T-cells. Current methods to enrich VSTs include the use of an IFN-γ capture system following peptide/antigen stimulation which lacks purity [106]. Alternatively, VSTs can be selected through the use of multimers, where HLA monomers loaded with viral peptides bind virus-specific TCRs [107]. Although stringent, this method is limited to single viral epitopes and is HLA-restricted, where only a subset of HLA-targeting multimers are commercially available. As such, deriving VSTs from iPSCs is an attractive approach, where the clonal replicative potential of iPSCs would enable the stringent selection of viral reactive clones. Coupled with the ability to generate HLA-specific iPSC cell lines, this could permit differentiation into polyclonal viral-reactive T-cells and is worthy of further study. A summary of the use of iPSCs in adoptive cell therapy is illustrated in Figure 4.

## 7. Potential and Challenges of iPSC-Derived Adoptive Cell Therapies

It is important to weigh the complexity and challenges of generating iPSC-based therapies against classical autologous and healthy-donor-derived allogeneic adoptive cell therapies. The manufacture of iPSC-derived adoptive cell therapies is costly/lengthy and does not shorten the time to treatment in the autologous setting. iPSCs hold more promise in the allogenic setting for their ability to generate multiple cell batches from single iPSC cell lines. However, can deriving cell therapies from iPSCs produce functionally superior cells to truly justify their use as autologous therapeutics? 

The production of classical NK cell therapies is challenging. NK cell therapies utilise NKs from peripheral/cord blood, where the proportion of NK cells is low at 10–20% and requires ex vivo expansion to obtain clinical doses [108]. Despite the ability to expand these cell populations, the cell yields are not sufficient for multiple patient dosing [109]. Moreso, the production of NK-CARs with multiple gene edits, as discussed above, introduces a number of technical challenges; namely, the transduction efficiency of primary and expanded NK cells can be highly variable and low [110,111]. Although the use of NK cell lines such as NK-92 can overcome some of these transduction and cell expansion challenges in vitro, for safety, they require irradiation prior to infusion, which restricts in vivo expansion/persistence, and clinical responses remain subpar [112]. In this setting, deriving NK cell therapies from iPSCs offers many advantages. The development of simple xenogeneic feeder-free manufactures consistently produces NK cells from iPSCs with comparable functionality to classical NK cells. This, accompanied by the clonal properties of iPSCs and their amenability to gene editing, offer an elegant solution for the standardised production of iPSC-derived NK cell therapies. There are many clinical trials underway to assess the safety and efficacy of multi-edited NK cell therapies, as per Table 2, which will inform their clinical potential. 

Unlike NK cells, the differentiation of T-cells from iPSCs is more complex. The current iPSC-to-T-cell differentiation methods involve several phases, as per Table 1 and the resulting cells vary considerably, which can lead to the emergence of erroneous/inconsistent T-cell populations with variable functions [32,66,67,68,76,81]. A priority for the field will be to improve standardisation and reduce the variability observed in these emerging iPSC-derived T-cell products. Despite the variable differentiation methods, studies demonstrate that T-cells derived from iPSCs appear rejuvenated. iPSC-derived HIV-specific T-cells were found to expand better after repeat stimulation and expressed markers of less differentiated Tcm subsets, including CCR7, CD27 and CD28, with elongated telomeres compared to paternal HIV-specific T-cells; however, their cytotoxicity was not compared to the parental cytotoxicity [32]. Similarly, in a feeder-free differentiation protocol, iPSC-derived TILs were found to have upregulated expression of Tcm markers, including CD62L, CD28 and TCF-7, increased telomere length, improved metabolic profile and enhanced expansion in vitro/in vivo against cancer spheroids when compared to parental TILs. iPSC-derived TILs demonstrate comparable in vitro cytotoxicity and inhibited spheroid engraftment in vivo compared to parental TILs; however, iPSC TILs also demonstrated non-specific cytotoxicity in non-cancer spheroids [102]. This rejuvenated profile, however, is not consistent across all studies; some demonstrate CD27/CD28 expression with the absence/low expression of CCR7/CD62L Tcm markers, whilst others lack expression at all [67,68,79]. Similarly, tumour control is either similar to parental therapies or lacking [67,68,102], where complete tumour regression was seen in parental CAR-Ts, but iPSC-derived CAR T-cells were only able to delay tumour regression [67]. It must be noted that not all iPSC-derived ACT studies compare their function to respective therapies manufactured via classical methods [34,79]. Moreso, particularly in the iPSC-derived CAR T-cell setting, in vivo models are established with far fewer tumour cells and larger T-cells doses accompanied, by the co-infusion of supportive cytokines including IL-2 and IL-15 [67,68]. Although IL-2 supplementation is frequently used in TIL therapy [113], it is not common practice in conventional CAR T-cell in vivo models or clinical therapy [93,114]. Such inconsistencies between studies and difficulty modelling long-term persistence in immunocompromised mouse models make it challenging to ascertain the true benefits of deriving T-cells from iPSCs versus the conventional manufacture of autologous CAR-T products for patients. 

The classical manufacture of adoptive T-cell therapies incorporating viral transduction and gene editing has been optimised over the years to produce highly functional T-cell therapies. This leads to the question of whether deriving T-cells from iPSCs provides additional advantages to justify their complex manufacture, and how tangible their potential towards clinical translation is. Whilst there have been significant advances in the development of GMP-appropriate, clinically applicable protocols for iPSC-based cell therapy manufacture that eliminate the use of xenogeneic feeder layers and serum [68,83], several unique challenges for clinical scalability remain. Firstly, such diverse/lengthy manufacturing harbours a significant cost burden. Secondly, tight control over the rapid generation, characterisation and long-term maintenance/stability of iPSC cell lines in clinical manufacturing is an area that still requires more development. Thirdly—and this is a critical quality control issue—is how the quantitative, qualitative and functional consistency of the cell populations derived between differentiation batches can be tested and controlled, as even simple variations in seeding density have been shown to affect lineage skew [115]. Lastly, several cell therapeutic approaches utilise genome-editing steps during the generation of ACTs, which poses further challenges with respect to product safety, as it is imperative that the field evolves towards nimble and low-cost approaches towards the identification of off-target effects and genome stability between batches. 

Some advances have been made towards the clinical translation of iPSC-derived cell therapeutics through the development of closed and automated bioreactor systems to support the controlled and large-scale manufacture of iPSC banks [116,117], as well as to support large scale differentiation [118]. Ultimately, significant collaboration between scientists, industry leaders and regulatory bodies is required to optimise clinically relevant protocols, develop resources to support the automation and standardisation of iPSC banking/differentiation, and develop regulatory pipelines for quality control and safety assessments of such iPSC-derived therapies.

Nonetheless, iPSCs possess huge potential, particularly in the allogeneic ACT setting where there are several iPSC-derived NK/CAR T-cell therapies currently in clinal trials, as illustrated in Table 2. Whilst the clinical efficacy of iPSC-derived T-cell therapies and their use, even in autologous ACT, remains to be determined, early phase I studies of iPSC-derived NK cell therapies have demonstrated clinical scalability and efficacy against a range of cancers [35,98], and their off-the-shelf nature makes them highly desirable in the ACT space. Despite progress, there remains a significant need for further development to optimize, enhance and standardise current iterations of iPSC-derived cell therapeutics; however, the use of iPSCs has already begun to pave a critical path as a source of next-generation adoptive cell therapies. 

## Figures and Tables

**Table 2:** **Registered iPSC−derived NK and T-cell therapy clinical trials.** This clinical trial list was compiled from those registered on ClinicalTrial.gov and the WHO Clinical Trials Registry.

**iPSC-Derived NK Cell Therapy**						
**Clinical Trial**	**Description**	**Condition**	**Company**	**Phase**	**Cell Product**	**Status**
**NCT03841110**	FT500 in combination with checkpoint inhibitors against solid tumours	Advanced solid tumour	Fate Therapeutics	I	iPSC-derived NK (allogenic)	Completed
**NCT04106167**	Long-term, non-interventional, observational study following treatment with Fate Therapeutics FT500	Cancer/Tumour	Fate Therapeutics	N/A	PSC-derived NK (allogenic)	Terminated
**NCT05182073**	FT576 in subjects with multiple myeloma (MM)	Multiple myeloma	Fate Therapeutics	I	iPSC-derived NK (allogenic)	Recruiting
**NCT04630769**	FT516 and IL2 with Enoblituzumab for ovarian cancer	Ovarian cancer; fallopian tube adenocarcinoma, primary peritoneal cavity cancer	Masonic Cancer Centre, University of Minnesota	I	iPSC-derived NK (non-cleavable CD16 Fc receptor) (allogenic)	Completed
**NCT04023071**	FT516 in combination with CD20-directed monoclonal antibodies	Advanced haematological malignancies	Fate Therapeutics	I	iPSC-derived NK cells (allogenic)	Terminated
**NCT04551885**	FT516 in combination with monoclonal antibodies	Advanced solid tumours	Fate Therapeutics	I	iPSC-derived NK	Terminated
**NCT04245722**	FT596 as a monotherapy and in combination with anti-CD20 monoclonal antibodies	B-cell lymphoma, chronic lymphocytic leukaemia	Fate Therapeutics	I	FT596 (hnCD16/anti-CD19 CAR/IL-15RF) iPSC-derived NK cells	Terminated
**NCT04555811**	FT596 with rituximab	Non-Hodgkin lymphoma, diffuse large B-cell lymphoma, high-grade B-cell lymphoma	Masonic Cancer Centre, University of Minnesota	I	(hnCD16/anti-CD19 CAR/IL-15RF) iPSC-derived NK cells	Active, not recruiting
**NCT05395052**	FT536 monotherapy and in combination with monoclonal antibodies	Advanced solid tumours	Fate Therapeutics	I	(hCD16/CD38KO/anti-MICA/B CAR/IL-15RF) iPSC-derived NK cells (allogeneic)	Terminated
**NCT05069935**	FT538 in combination with monoclonal antibodies	Advanced solid tumours	Fate Therapeutics	I	FT538 (hnCD16/CD38KO/IL-15RF) iPSC-derived NK cells	Terminated
**NCT04714372**	FT538 in combination with daratumumab	Acute myeloid leukaemia	Masonic Cancer Centre, University of Minnesota	I	FT538 (hnCD16/CD38KO/IL-15RF) iPSC-derived NK cells	Recruiting
**NCT04614636**	FT538	Advanced hematologic malignancies	Fate Therapeutics	I	FT538 (hnCD16/CD38KO/IL-15RF) iPSC-derived NK cells	Terminated
**IRCT20200429047241N1**	Personalized immunology of patients with advanced breast cancer using induced pluripotent stem cell-derived natural killer cells	Breast cancer	Tehran University of Medical Science	I	Autologous iPSC-derived NK cells	Recruiting
**iPSC-Derived T-cell Therapy**						
**NCT05336409**	CNTY-101	CD19-positive B-cell malignancies (ELiPSE-1)	Century Therapeutics, Inc.	I	(sIL-15/EGFRt/anti-CD19 CAR) iPSC-derived T-cells with IL-2	Recruiting
**NCT04629729**	FT819	B-cell malignancies	Fate Therapeutics	I	iPSC-T (CAR-19, TCR-KO)	Recruiting
**NCT03407040**	**Generation of cancer antigen-specific T-cells from human induced pluripotent stem cells (iPSC) for research and potential future therapy**	**Gastrointestinal cancers, breast cancer, pancreatic cancer, melanoma, lung cancer**	**National Cancer Institute (NCI)**	**N/A**	**Cancer antigen-specific T-cells from human induced pluripotent stem cells**	**Terminated**

## Abbreviations

aAPC	Artificial antigen-presenting cell
ACT	Adoptive cell therapy
ADCC	Antibody-dependent cellular cytotoxicity
Adv	Adenovirus
AICD	Activation-induced cell death
AP	Alkaline phosphatase
APC	Antigen-presenting cell
ATO	Artificial thymic organoids
BCMA	B-cell maturation antigen
bFGF	Basic fibroblast growth factor
BMP-4	Bone morphogenetic protein 4
CAR-T	Chimeric antigen receptor T-Cell
CMV	Cytomegalovirus
CRS	Cytokine release syndrome
CXCR	Chemokine receptor
DAG	Diacylglycerol
DLL	Delta-like ligand
DN	Double negative
DP	Double positive
EB	Embryoid body
EBV	Epstein–Barr virus
EHT	Endothelial-to-hematopoietic transition
EMO	Embryonic mesodermal progenitor
ESC	Embryonic stem cell
FDA	Food and Drug Administration
FLT3L	Fms-related tyrosine kinase 3 ligand
GPC-3	Glypican-3
GvHD	Graft-versus-host disease
HDR	Homology-directed repair
hEMP	Human embryonic mesoderm progenitor
HIV	Human immunodeficiency virus
HLA	Human leukocyte antigen
HPV16	Human papilloma virus type 16
HSC	Hematopoietic stem cell
IFN	Interferon
IL	Interleukin
iPSC	Induced pluripotent stem cell
ISP	Intermediate single positive
mb	Membrane-bound
MHC	Major histocompatibility complex
MM	Multiple myeloma
MOI	Multiplicity of infection
NK	Natural killer
Oct	Octamer-binding transcription factor
PBMC	Peripheral blood mononuclear cells
PD-1	Programmed cell death protein 1
PHA	Phytohemagglutinin
RAG	Recombination-activating gene
Rb	Retinoblastoma
REP	Rapid expansion protocol
SCF	Stem cell factor
scFv	Single-chain variable fragment
SDF-1α	Stromal cell-derived factor 1 alpha
shRNA	Short hairpin RNA
SNP	Single-nucleotide polymorphism
Tcm	Central memory T-cell
TCR	T-cell receptor
Te	Effector T-cell
Th	T helper
TIL	Tumour-infiltrating lymphocyte
TMG	Tandem minigene
Tn	Naïve T-cell
TPO	Thrombopoietin
TRAC	T-cell receptor α constant
Tte	Terminal T-cell
VEGF	Vascular endothelial growth factor
VST	Viral-specific T-cell
WGS	Whole-genome sequencing
